# Development of Bioactive Edible Film and Coating Obtained from *Spirogyra* sp. Extract Applied for Enhancing Shelf Life of Fresh Products

**DOI:** 10.3390/foods14050804

**Published:** 2025-02-26

**Authors:** Siriwan Soiklom, Wipada Siri-anusornsak, Krittaya Petchpoung, Sumpan Soiklom, Thanapoom Maneeboon

**Affiliations:** 1 Scientific Equipment and Research Division, Kasetsart University Research and Development Institute (KURDI), Kasetsart University, Bangkok 10900, Thailand; wipada.s@ku.th (W.S.-a.); rdikyp@ku.ac.th (K.P.); rditpm@ku.ac.th (T.M.); 2 Department of Industrial Chemistry, Faculty of Applied Science, King Mongkut’s University of Technology North Bangkok, Bangkok 10800, Thailand; sumpan.s@sci.kmutnb.ac.th

**Keywords:** antioxidant, bioactive coating, cytotoxicity, edible films, *Spirogyra* sp., shelf-life extension

## Abstract

The growing interest in environmentally friendly food packaging has led to the development of bio-edible alternatives. This study developed novel, edible, active films and coatings to enhance the shelf life of fresh products. Crude bioactive algal extract (CBAE) was obtained from the ethanol extraction of *Spirogyra* sp. and incorporated into chitosan-based films and coatings at varying concentrations. The CBAE was rich in phenolic compounds and had antioxidant activity and potential antibacterial properties. The films were prepared using a solvent-casting method and characterized for their biochemical and physical properties. The incorporation of CBAE enhanced the antioxidant activity and improved the tensile strength of the films by 80%. Additionally, film transparency and water vapor permeability were reduced by 13% and 50%, respectively, compared to the control. The developed CBAE coating solution exhibited biocompatibility with human colon adenocarcinoma (HT-29) and mouse subcutaneous connective tissue (L929) fibroblast cells. A shelf-life evaluation using a coating-dipping method on okra showed that the CBAE-coated samples maintained better weight retention and firmness than the uncoated samples over 5 days of storage at ambient temperature. Based on these findings, the CBAE-based edible films and coatings could serve as sustainable alternatives for extending the shelf life of fresh products.

## 1. Introduction

The current increasing demand by health-conscious consumers for fresh, high-quality food products has resulted in a growing preference for packaging materials that are made from natural products and are natural and environmentally friendly. Consumers are now seeking packaging that not only preserves the quality of the food but also incorporates health-promoting compounds, such as antioxidants, fiber, and antimicrobials, to enhance both mental and physical well-being. This shift in consumer behavior is further driven by concerns about food waste, environmental degradation, and the depletion of natural resources that all result in major economic losses. Active, edible packaging offers a sustainable alternative to conventional plastic packaging and is gaining traction as a solution to reduce food spoilage during storage. These innovative non-plastic materials incorporate plant extracts and other bioactive compounds, providing functional benefits such as antioxidant and antimicrobial properties. Beyond enhancing food preservation, edible packaging also minimizes waste, helping to mitigate the environmental hazards associated with plastic packaging. The use of edible biopolymers, as opposed to traditional plastic, has become increasingly desirable. Edible biopolymers not only improve food safety and extend product shelf life but also offer an environmentally friendly option that has no detrimental impact on human health [1].

Chitosan (CS), a natural biopolymer derived from chitin, is widely used in edible food packaging due to its non-toxic, biodegradable, and film-forming properties [2]. In addition, CS is cost-effective because it is readily available from natural sources. Notably, the polycationic structure of chitosan has been recognized for its antibacterial and antifungal properties, making it a promising material for food preservation [3,4,5]. Despite its benefits, chitosan films have limitations, such as high water vapor permeability. Since moisture control and gas barrier properties are essential for food packaging, these limitations reduce the functionality of chitosan-based films in certain applications [6]. Researchers have sought to improve the physicochemical and structural properties of chitosan films by incorporating various bio-fillers, such as neutral lipids, fatty acid waxes, proteins, polysaccharides, and plant extracts [7,8]. Algae have been identified as an increasingly promising source of biopolymers for enhancing edible film properties. Algae are abundant in nature, highly nutritious, and contain a range of bioactive compounds known for their health benefits [9]. Biopolymers derived from algae, such as alginate, carrageenan, and agar, are being explored for use in food packaging due to their unique properties, including biocompatibility, high water retention, and the ability to form strong, flexible films [10]. Algal materials are non-toxic, biodegradable, and offer a sustainable alternative to synthetic food packaging materials [11]. In addition to their environmental and functional benefits, algal-based films have important nutritional value, as well as excellent antioxidant and antimicrobial properties, making them ideal candidates for food packaging [12,13]. *Spirogyra* sp., a freshwater green alga, locally known as “Tao” in Northern Thailand, is traditionally consumed for its rich nutritional composition. It is particularly noted for its bioactive compounds, which contribute to its antioxidant and antimicrobial effects [14,15]. The unique properties of algal-based films, such as their resistance to fats and oils, high water retention capacity, and antioxidant activity, make them well suited for preserving food quality during storage [16,17]. While chitosan-based films have been studied extensively, they often have poor mechanical strength and barrier properties, limiting their use as effective packaging materials [18]. To overcome these shortcomings, incorporating algal biopolymers into CS films has shown promise in enhancing their functional properties, with studies demonstrating that the addition of algal extracts can improve the mechanical strength, water barrier properties, and antioxidant activity of CS films [19,20]. For example, edible films made from CS combined with extracts from brown macroalgae have been reported to have high antioxidant activity [21]. Zaghbib et al. (2025) showed that incorporating *Ulva lactuca* extract into CS films enhanced their antioxidant and antibacterial properties [22]. Karkar et al. (2023) further demonstrated that the addition of *Nigella sativa* extract improved the mechanical properties of CS-based films [4]. However, the effect of *Spirogyra* sp. extract on edible films and coatings remains largely unexplored.

The objective of the current study was to develop a highly functional and sustainable CS-based, edible, bio-composite film and coating, incorporating bioactive crude extract from *Spirogyra* sp. The aim was to use crude bioactive algal extract (CBAE) as a bio-filler to enhance the performance of these edible films, particularly their antioxidant, antibacterial, and preservative properties. The results of the current research should contribute to the creation of more effective and eco-friendly food packaging materials. Additionally, the study evaluated the ability of these bioactive films and coatings to preserve the physicochemical properties of fresh produce, specifically okra (*Abelmoschus esculentus*), with the goal of extending its shelf life.

## 2. Materials and Methods

### 2.1. Chemical

CS was obtained from Sigma-Aldrich (USA). 2,2-Diphenyl-1-picrylhydrazyl (DPPH), gallic acid, Folin-Ciocalteu phenol reagent, 2,2′-azino-bis (3-ethylbenzothiazoline6-sulfonic acid, and 2,4,6-tri(2-pyridyl)-s-triazine were purchased from Sigma-Aldrich (Saint Louis, MO, USA). Ethanol was sourced from J.T. Baker (Phillipsburg, NJ, USA).

### 2.2. Preparation of Bioactive Crude Algae Extract

Samples of the dried green alga *Spirogyra* sp. (Tao) were obtained from Nan province, Thailand, and transported to the Scientific Equipment and Research Division, Kasetsart University, Bangkok, Thailand. The algal samples were ground and passed through a 40-mesh sieve. In total, 50 g of ground algae were soaked in 500 mL of ethanol using stable conditions for 24 h. Then, the extraction process was enhanced using ultrasound-assisted extraction for 30 min, with an ultrasonic bath (GT-1860QTS; GT Sonic; Shenzhen, Guangdong, China) at a frequency of 40 kHz. Subsequently, the extract was passed through Whatmam^TM^ No 5 filter paper. The crude bioactive algal extract (CBAE) was stored in a dark container at 4 °C until further analysis.

### 2.3. Bioactive Analysis

#### 2.3.1. Total Phenolic Content (TPC) Determination

TPC determination was performed using a modified Folin–Ciocalteu method, based on Ceymann et al. (2012) [23]. Briefly, 1.25 mL of 1 N Folin–Ciocalteu’s reagent was added to 0.5 mL of the sample, then 1.25 mL of a sodium carbonate solution (10% *w*/*v*) was added and mixed well. The absorbance was measured at 765 nm using a spectrophotometer (UV-1900; Shimadzu; Kyoto, Japan) after 30 min incubation periods. The TPC was calculated as milligrams of gallic acid equivalent (GAE) per gram of sample.

#### 2.3.2. Antioxidant Activity Measurement

- 2,2-Diphenyl-1-picrylhydrazyl (DPPH) Radical Scavenging Activity

The DPPH radical scavenging activity was determined using a modified method by Vieira et al. (2011) [24]. Briefly, each sample was added to 0.1 mM DPPH in methanol and left in a dark place under ambient conditions. After 30 min of incubation, the solution was measured at 517 nm. The DPPH radical scavenging activity was calculated as milligrams of gallic acid (GAE) equivalent per gram of sample.

- Ferric Reducing Antioxidant Power (FRAP) Determination

A modified FRAP assay was performed, based on Benzie and Strain (1996) [25]. The FRAP reagent was prepared by mixing 1 part of 10 mmol/L 2,4,6-tri(2-pyridyl)-s-triazine, 10 parts of 300 mmol/L sodium acetate buffer (pH 3.6), and 1 part of 20 mmol/L ferric chloride. Then, 1.5 mL of FRAP reagent was added to 50 μL of the sample, incubated at room temperature for 4 min, and measured at 593 nm using a spectrophotometer (UV-1900; Shimadzu; Kyoto, Japan). The FRAP activity was calculated in terms of milligrams of gallic acid (GAE) equivalent per gram of sample.

- ABTS Radical Scavenging Activity

A modified 2,2′-azino-bis (3-ethylbenzothiazoline6-sulfonic acid) ABTS was performed, based on Márquez-Reyes et al. (2022) [26]. A total of 1.0 mL of ABTS reagent was added to 0.5 mL of sample. After 45 min incubation in the dark at ambient temperature, the solution was measured at 734 nm using a spectrophotometer (UV-1900; Shimadzu; Kyoto, Japan). The ABTS radical scavenging activity was determined as milligrams of gallic acid (GAE) equivalent per gram of sample.

#### 2.3.3. Chlorophyll Determination

The chlorophyll concentrations were determined, according to the method described by Godlewska et al. (2017) [27]. The chlorophyll content of the crude bioactive algae extract (CBAE) was measured at wavelengths of 663 nm and 645 nm using a spectrophotometer (UV-1900; Shimadzu; Kyoto, Japan), and the total chlorophyll was calculated using the equation:

Total Chlorophyll=8.02×A(663)+20.2×A(645)

where A(663) and A(645) are the absorbances measured at 663 nm and 645 nm, respectively.

### 2.4. Microbiological Analysis

The antimicrobial activity of the CBAE was assessed using the broth microdilution method to determine the minimum inhibitory concentration (MIC) against *Aspergillus niger, Staphylococcus aureus* DMST 8840, and *Escherichia coli* DMST 4212; bacterial strains received from the Department of Medical Science, Ministry of Public Health, Thailand, according to the method of Basri et al. (2005) [28]. For the assay, serial two-fold dilutions of the CBAE were prepared in a Mueller–Hinton broth for bacteria and potato dextrose broth for fungi. Each well was inoculated with 100 µL of microbial suspension (adjusted to a concentration of approximately 1.0 × 10^8^ CFU/mL for bacteria and 1.0 × 10^6^ spores/mL for fungi). The plates were incubated at 37 °C for 24 h for bacterial strains and at 30 °C for 72 h for fungal strains. The MIC was determined as the lowest concentration of the CBAE that inhibited visible microbial growth, which was observed by the absence of turbidity for bacteria or visible growth for fungi. Negative controls (broth only) and positive controls (appropriate antibiotics or antifungals) were included in each test.

### 2.5. Active Edible Films and Coatings Formation

A solution casting technique was chosen for film preparation. Briefly, 1 g of chitosan (CS) was dissolved in 0.3% (*w*/*v*) acetic acid. Then, the CBAE at amounts of 0%, 5%, 10%, 15%, 20%, 25%, and 30% (*v*/*v*) were added to the CS solution. Each mixture was stirred until homogeneous and left at 4 °C for 24 h to eliminate air bubbles. Next, each film solution was spread onto silicone plates (11 × 20 cm) and dried under ambient conditions (28.5 ± 2 °C, 60–70% RH) for 36 h. All film samples were stored in a dry closed box under ambient conditions of 27.0 ± 2 °C and 60–70% RH.

### 2.6. Film Evaluation

#### 2.6.1. Transparency of Active Edible Films

Film transparency was performed, following the modified method of Qin et al. (2015) [29]. A film sample (0.7 × 1.5 cm) was placed in the spectrophotometer. The absorbance was measured at 600 nm, with an empty test cell as a reference. Transparency was ex-pressed as a percentage.

#### 2.6.2. Fourier Transform Infrared (FTIR) Spectroscopy

FTIR spectroscopy was performed to analyze the functional groups present in CBAE and the chitosan-based films. The spectra were obtained using an FTIR spectrometer (PerkinElmer Spectrum100; PerkinElmer; Shelton, CT, USA). The wavenumber range for the analysis was 4000–500 cm⁻^1^ with a resolution of 4 cm^−^^1^. The samples were prepared by placing a small amount of the dried extract or film in the form of a pellet with potassium bromide (KBr) and analyzed in transmission mode.

#### 2.6.3. Scanning Electron Microscopy (SEM) Analysis

The film surface morphology was determined using a Quanta 450 FEI scanning electron microscope (Thermo Fisher Scientific; Hillsboro, OR, USA) at 10 kV under a low vacuum mode at 2000× magnifications.

#### 2.6.4. Determination of Mechanical Properties

The tensile strength was measured using a TA-XT texture analyzer (TA-HD Plus; Stable Micro System; Godalming, Surrey, UK) on 50 × 10 mm film samples with a film thickness of 0.01 mm (approximately 0.01 g).

#### 2.6.5. Water Vapor Permeability (WVP) Analysis

The WVP was determined, according to modifications to the method of ASTM [30]. Film samples with a 0.01 mm thickness were cut, mounted, sealed, and placed over a water container in a chamber maintained at 80% RH using saturated sodium chloride. Once equilibrium conditions had been achieved, eight weight measurements were recorded.

#### 2.6.6. Antimicrobial Test of Bio-Composite Films

Antimicrobial activity testing of the bio-composite films was carried out using the agar diffusion method according to the modified method of Chana-Thaworn et al. (2011) [31]. Briefly, films of 16 mm diameter discs were placed on the previous smear Mueller–Hinton agar surface inoculated with *S. aureus* DMST 8440 (approximately 1.0 × 10^6^ CFU/mL of tested bacteria). The zone of inhibition was observed after being incubated at 37 °C for 24 h.

### 2.7. Shelf-Life Study of Okra (Abelmoschus esculentus)

#### Edible Coating Application

Fresh okra was purchased from a local market in Thailand. Samples were selected, with uniform size and color maturity and without microbiological contamination, and then washed and dried under ambient conditions (27 ± 2 °C, 60–70% RH). Each sample was dipped in the coating solution for 1 min, rested for 3 min, with this step being repeated three times. The coated samples were left under ambient conditions (27 ± 2 °C, 60–70% RH). Weight loss, color, and firmness were recorded to determine the effect of coating on the okra.

### 2.8. Coated Okra Characterization

#### 2.8.1. Weight Loss Determination

The weight loss content was determined based on the modification method of Borges et al. (2023) using an analytical weighing balance (ATY224R; Shimadzu; Kyoto, Japan) [32]. The samples were stored under ambient conditions (27 ± 2 °C, 60–70% RH) for 5 days and then each sample (approximately 20 g) was determined for weight loss. The weight loss results were calculated using the equation:

$$\mathrm{Weight}\;\mathrm{loss}\;(\%)=\frac{(Wt0-Wtd)\times 100}{Wt0}$$

where Wt0 is the initial weight of the okra sample and Wtd is each daily weight of the okra sample.

#### 2.8.2. Color Measurement

The color of the samples was measured using a colorimeter (HunterLab; Ultrascan Pro; Restion, VA, USA).

#### 2.8.3. Firmness and Toughness

Each sample (approximately 20 g) was evaluated for firmness and toughness using a textural analyzer (TA-HD Plus; Stable Micro System; Godalming, Surrey, UK) at three random points per sample. Firmness and toughness were reported as the force in newtons (N) and the force in newton seconds (N.s), respectively.

### 2.9. Cytotoxicity Study

The mitochondrial activity of human colon adenocarcinoma (HT-29) cells and mouse subcutaneous connective tissue (L929) cells cultured in the sample solutions was determined using tetrazolium dye, 3- (4,5-dimethylthiazol-2-yl)-2,5- diphenyltetrazolium bromide (MTT) enzymatic conversion, according to Mosmann (1983) [33]. Briefly, HT-29 and L929 were cultured using Dulbecco’s modified eagle’s medium and incubated in a 5% CO_2_ humidified atmosphere at 37 °C for 24 h. After discarding the seeding, 100 µL samples were added to plates and placed under the same conditions as above. Then, 100 µL of 0.5 mg/mL MTT solution was added after removing the samples and further incubated using the same conditions as above for 24 h. Subsequently, 100 µL of DMSO was added after discarding the previous MTT solution and the solution was incubated under the same conditions as above for 10 min. Cell viability was evaluated using a 96-well microplate reader (Biochrom; Melbourne, Australia) at 570 nm.

### 2.10. Data Analysis

The analysis was based on data from a completely randomized design consisting of three replicates. Data were presented as mean ± standard deviation (SD) values. Statistical analysis was carried out using the SPSS version 14.0 for Windows software (IBM Corp; Armonk, NY, USA).

## 3. Results

### 3.1. Biochemical and Microbiological Analysis

Before being used for film and coating preparation, the biochemical properties of the CBAE were analyzed, consisting of antioxidant activity, TPC, and chlorophyll. Based on the results, the CBAE was rich in antioxidants with DPPH high levels of antioxidant activity (2.15 mg GAE/g), FRAP activity (14.59 mg GAE/g), TPC (1.10 mg GAE/g), and total chlorophyll (61.64 mg/100 g), as shown in Table 1. These results indicate that the extract has potential as an antioxidant compound, which aligns with other reports on the antioxidant capacity of *Spirogyra* sp. [14,34,35,36,37].

The CBAE solution had antimicrobial activity, inhibiting the growth of *A. niger* (MIC = 25 mg/mL), *S. aureus* (MIC = 6.25 mg/mL), and *E. coli* (MIC = 25 mg/mL). These findings were comparable to other reports on the antimicrobial potential of *Spirogyra* sp. against bacterial strains [38,39,40].

### 3.2. Flim Characterization

#### 3.2.1. FTIR Analysis

**Figure 1:**
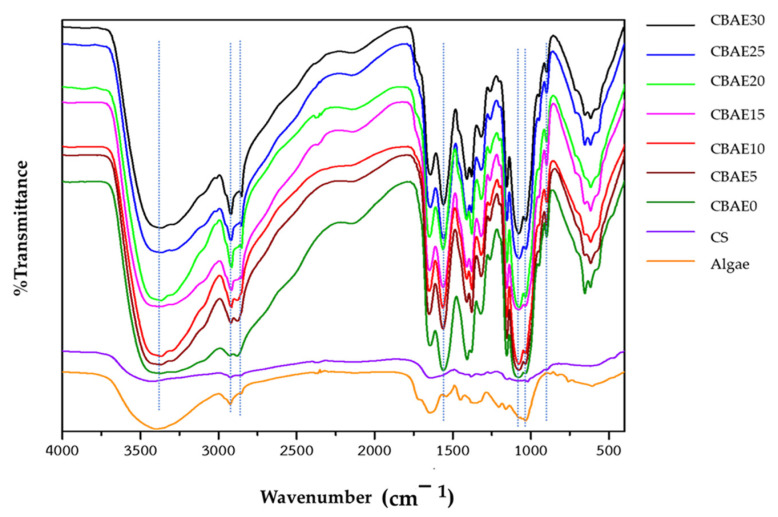
FTIR spectra of CBAB and bio-composite films, where CS = chitosan powder, CBAE0 = CS without CBAE, CBAE5 = CS with 5% CBAE, CBAE10 = CS with 10% CBAE, CBAE15 = CS with 15% CBAE, CBAE20 = CS with 20% CBAE, CBEA25 = CS with 25% CBAE and CEAE30 = CS with 30% CBAE.

The FTIR spectrum is an important qualitative key function for characterizing functional groups and their corresponding frequencies present on their surface materials. The FTIR spectrum of CS (Figure 1) showed a broad peak at 3400 cm^−^^1^, resulting from –NH and O–H stretching overlapping the chitosan structure, while the peak at 1548 cm^−^^1^ corresponded to N–H bending of amines. In addition, absorption peaks related to CH_2_ bending and C–O stretching were visible in the 1406 cm^−^^1^ and 1090 cm^−^^1^ regions. The FTIR spectrum of the algal powder showed a broad overlapping of O–H stretching with N–H stretching in the 3400 cm^−^^1^ regions. A–CH stretching vibration of the group of polyphenols was evident at 2970 cm^−^^1^. Peaks at 1654 cm^−^^1^ corresponded to the N–H of amide. Furthermore, the peak at 1200–1300 cm^−^^1^ belonged to –CH_2_ vibration and at 1045 cm^−^^1^ could have been due to the presence of polysaccharides. The FTIR spectrum of the algal film revealed a peak of functional groups similar to those found in CS and algae, with a peak of 2970 cm^−^^1^ that correlated with the –CH stretching vibration of the polyphenols. In addition, a 1200–1300 cm^−^^1^ peak was found, indicating –CH_2_ vibration of the amide in addition to a peak at 1087 cm^−^^1^, which corresponded to polysaccharides. The peaks in the 900 cm^−^^1^ region represented vibrational stretching of the –CH_2_ of ethanol. However, all the CBAE film and algal extracts had a prominent peak at 2900 cm^−^^1^. No major differences were observed in the FTIR spectra of the algal films at various concentrations.

#### 3.2.2. Film Surface Morphology

**Figure 2:**
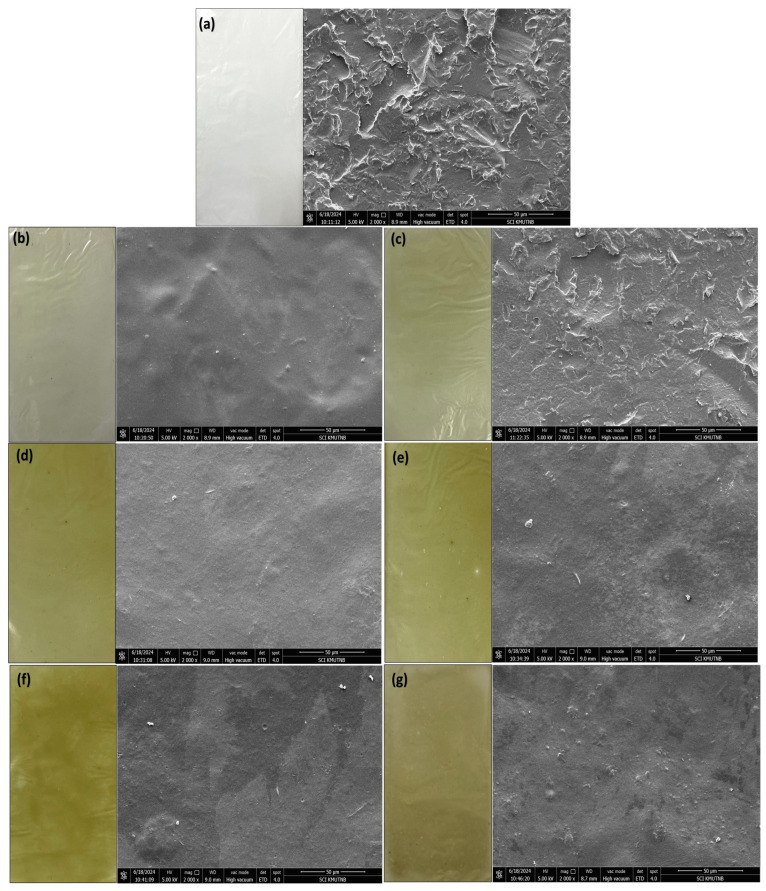
SEM images (**right**) and digital photographs (**left**) of films at 2000× magnification: (**a**) chitosan film (CS); (**b**) CS with 5% CBAE; (**c**) CS with 10% CBAE; (**d**) CS with 15% CBAE; (**e**) CS with 20% CBAE; (**f**) CS with 25% CBAE; and (**g**) CS with 30% CBAE.

The surface characteristics of the coated films are presented in Figure 2. The control film had a smooth, compact surface without pores or cracks compared to the bio-composite films. The increase in the CBAE in the bio-composite films led to irregular, heterogeneous, and rougher surfaces than in the control. Additionally, the higher the CBAE load was, the more small particles were detected on the film surface. These particles could be attributed to the presence of bioactive molecules from the CBAE, such as polyphenols, proteins, and polysaccharides, which may have contributed to the roughness and increased the adhesion properties of the film. A similar phenomenon of surface roughness has been reported in other studies [41,42,43]. This effect could be attributed to the presence of bioactive molecules, including polyphenols, proteins, and polysaccharides [41,42,43,44].

#### 3.2.3. Water Vapor Permeability of Developed Films

**Figure 3:**
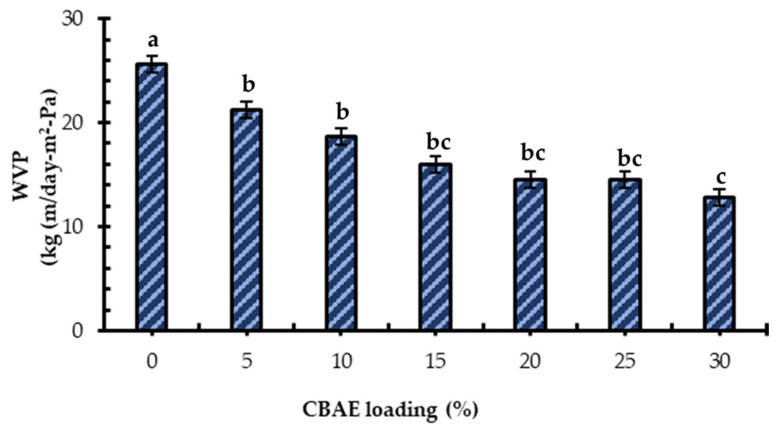
Effect of CBAE loading on the water vapor transmission rate of the CS-based edible films. ^a,b,c^ = different lowercases letters for CBAE loading indicate significant (*p* < 0.05) differences and error bars indicate ± SD.

Avoiding or reducing the moisture transfer in food and its surroundings is preferred for food packaging; thus, the WVP of the film should be as low as possible [45]. In the current study, the WVP value of the developed edible bio-composite films significantly reduced with increasing CBAE concentrations (Figure 3). Compared to the control, the highest reduction in WVP (approximately 50%) was in the bio-composite film containing 30% CBAE, which indicated that the packaging would have a longer shelf life. These results agree with those of Martin et al. [46], who studied edible films incorporated with proteins and bioactive compounds. The improvement in the WVP may have been due to the formation of new interactions between the electronegative atoms (such as oxygen) in the bio-filler (CBAE) and the hydrogen atoms in the CS, which led to the formation of new hydrogen bonds and enhanced the water vapor barrier properties of the film.

#### 3.2.4. Color Parameters

**Figure 4:**
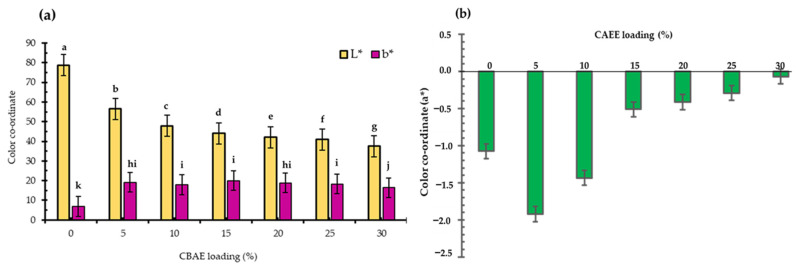
Effect of CBAE loading on color parameters of CS-based edible films: (**a**) L* and b*; and (**b**) a*, where ^a,b,c,d,e,f,g,h,i,j,k^ = different lowercase letters in CBAE loading indicate significant (*p* < 0.05) differences and error bars indicate ± SD.

Figure 4 presents the color coordinates in terms of L* (lightness), b* (yellowness), and a* (greenness) of the developed bio-composite films, as determined using a Hunter colorimeter. Significant differences (*p* < 0.05) were observed in the L*, a*, and b* values between the bio-composite films and the control. In general, as the CBAE concentration increased, the L* and a* values decreased as the b* value increased (Figure 4a). The observed negative a* value suggests the existence of chlorophyll. The highest negative a* value was recorded in the film containing 30% CBAE (Figure 4b). These findings might have been due to the derivatized pigments, such as carotenoids, formed during the process. In addition, the effect has been reported of natural-colored pigments on the color coordinates of active films [47]. This was in agreement with Kumar et al. (2021), who reported that adding pomegranate peel extract into CS-based films improved color properties [3].

#### 3.2.5. Transparency of Films

**Figure 5:**
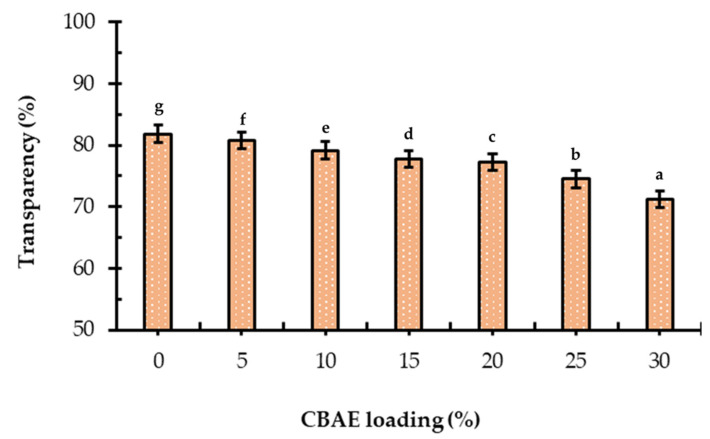
Effect of CBAE loading on transparency of CS-based edible films, where ^a,b,c,d,e,f,g^ = different lowercase letters for CBAE loading indicate significant (*p* < 0.05) differences and error bars indicate ± SD.

Transparency is a critical property in film packaging, influencing the visual appeal of the packaged food products. The transparency of the incorporated CBAE-films significantly (*p* < 0.05) decreased with an increasing CBAE concentration (Figure 5). The film with 30% CBAE had the lowest transparency due to the presence of active compounds in the extract. These results suggest that incorporating CBAE into the CS matrix reduced the light transmittance of the films, resulting in improved blocking of light, which was consistent with other studies, where the incorporation of pomegranate peel extract into CS-based films reduced the transparency due to the presence of hydrolyzed tannins [29].

#### 3.2.6. Antioxidant Activity and Total Phenolic Content

**Figure 6:**
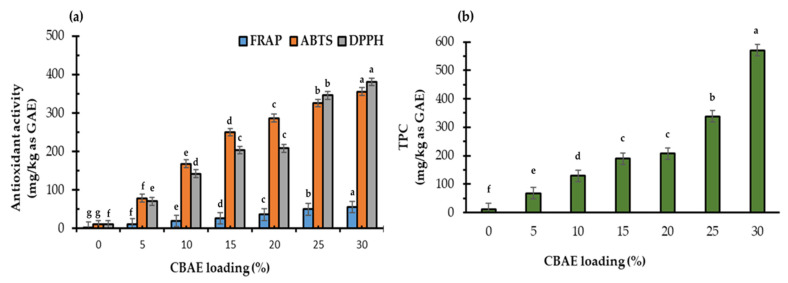
Effect of CBAE loading on (**a**) antioxidant activity and (**b**) TPC of CS-based edible films, where ^a,b,c,d,e,f,g^ = different lowercase letters with CBAE loading indicate significant (*p* < 0.05) differences and error bars indicate ± SD.

Figure 6 presents the percentage of antioxidant activity and total phenolic compounds of the films coated with CBAE. The antioxidant activities (DPPH, FRAP, and ABTS assay) and TPC significantly (*p* < 0.05) increased in proportion to the CBAE loading (Figure 6a,b). These phenomena might indicate the appearance of bioactive compounds, such as phenolics, tannins, and flavonoids, in the bio-composite films. The highest antioxidant activity and TPC levels were in the 30% CBAE bio-composite film, with a 20-fold increase in antioxidant activity compared to the control (Figure 6b). These increases in antioxidant activity could maintain the properties of goods such as texture, color, and freshness [48]. Likewise, these results were in agreement with Wang et al. (2013), who reported improved antioxidant properties in CS-based bio-composite films enriched with polyphenols [49].

#### 3.2.7. Antimicrobial Result of Bio-Composite Films

**Figure 7:**
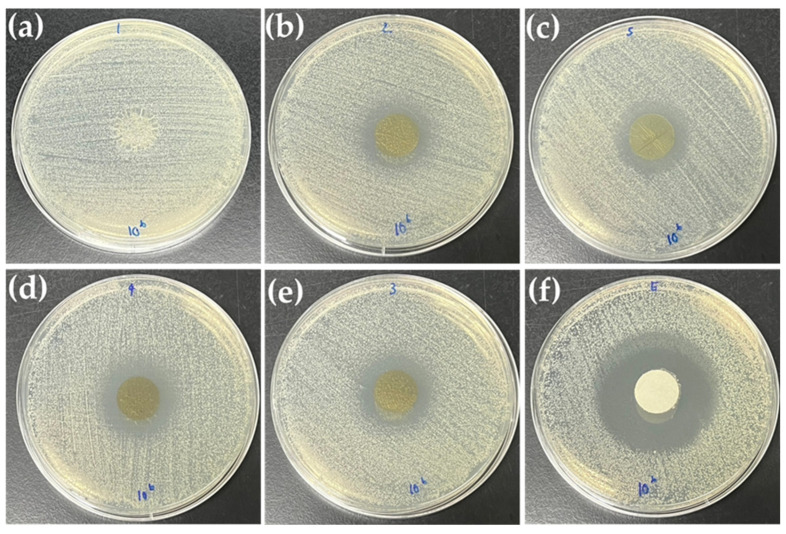
Antimicrobial activity of the CS-based edible films, where (**a**) = chitosan film (CS); (**b**) = CS with 5% CBAE; (**c**) = CS with 20% CBAE; (**d**) = CS with 25% CBAE; (**e**) = CS with 30% CBAE; (**f**) = positive control (Erythromycin, 15 µg/mL).

The antibacterial activity against Gram-positive *S. aureus* was evaluated for incorporated CBAE-films. The visible inhibition zones against bacteria of incorporated CBSE-films increased with the increase in CBAE concentration compared with the control (Figure 7). There were significant differences (*p* < 0.05) in the mean diameter inhibition zone of bio-composite films. The incorporated CBAE-films with 30% CBAE showed high activity against *S. aureus* (22 mm), and the lowest microbial activity was found at 5% CBAE incorporated CBAE-films (18 mm), which is lower than the positive controls (42 mm). The increase in CBAE could enhance microbial activity. The appearance of inhibition zones in the samples confirmed the antimicrobial activity. The results agreed with Chana-Thaworn et al. (2012) that kiam wood extract-incorporated films promoted microbial activity [31].

#### 3.2.8. Mechanical Properties

**Figure 8:**
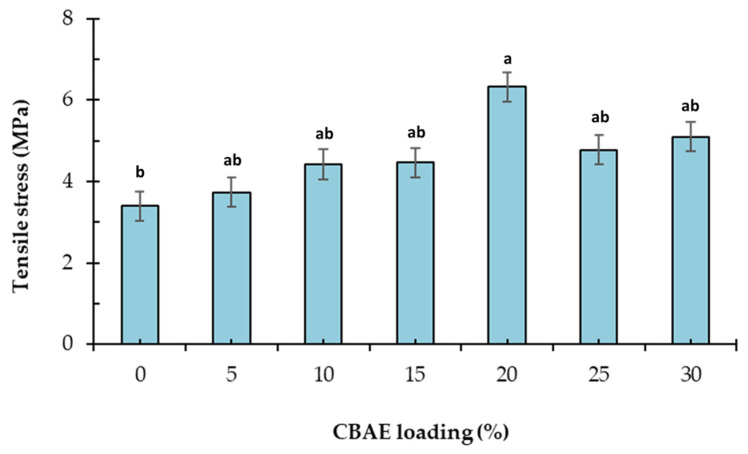
Effect of CBAE loading on tensile strength of CS-based edible films, where ^a,b^ = different lowercase letters with CBAE loading indicate significant (*p* < 0.05) differences and error bars indicate ± SD.

One of the most important elements in coating and packing products in edible films is tensile strength. Tensile strength is responsible for film flexibility. Films with lower tensile strength show more flexibility than those with higher tensile strength [50]. The tensile strength value of the films was 3.1–6.3 MPa (Figure 8). The addition of CBAE to the film resulted in a significant increase in tensile strength up to a 20% CBAE concentration. Beyond this concentration, a slight decrease in tensile strength was observed. Coated film at a 20% CBAE concentration had the highest tensile strength value as, without an added CBAE film, it expressed the lowest. Furthermore, the flexibility film reduction could be caused by the mobility chain of strong interaction between hydrogen bonding of the CBAE and polymer matrix [51]. These data are comparable with Biratu et al. (2024) of coffee pulp films [52].

### 3.3. Biocompatibility of the Formulated Active Coating Solution

**Figure 9:**
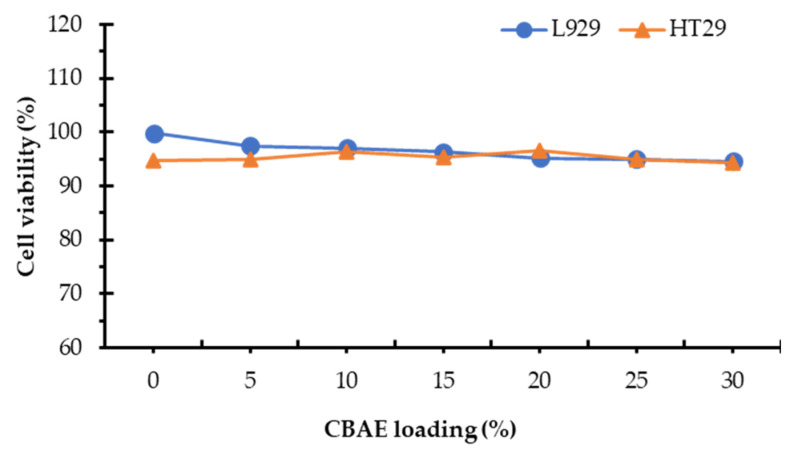
Effect of CBAE loading on cell viability of the active coating solution.

**Figure 10:**
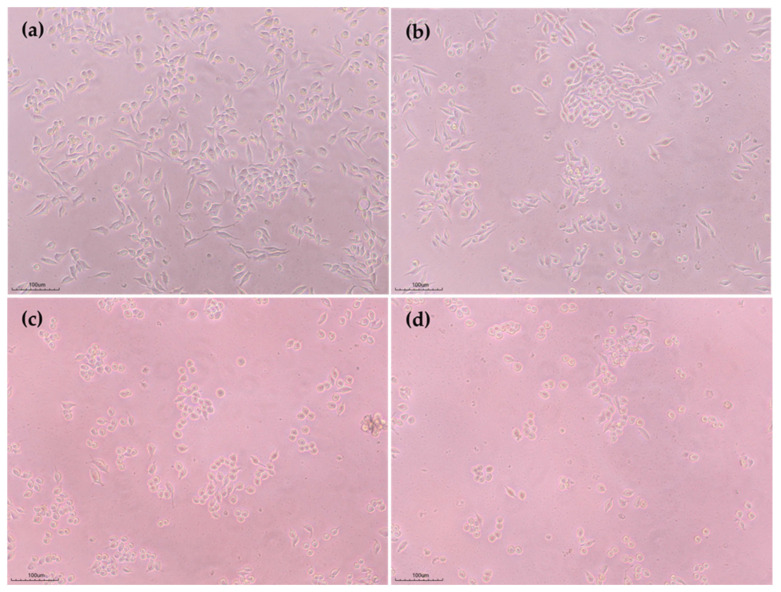
Morphological changes in HT29 and L929 cells observed under inverted light microscope (Nikon ECLIPSE TS100) at 100× magnification after incubation in medium for 24 h: (**a**) CS (tested with HT29); (**b**) CS with CBAE (tested with HT29); (**c**) CS (tested with L929) and (**d**) CS with CBAE (tested with L929).

Cytotoxicity materials used in healthcare must undergo biological tests to assess their safety and risk, with a material being considered cytotoxic if the cell viability is below 70% compared to untreated cells [53]. Figure 9 shows the results of the biocompatibility test based on the MTT assay to evaluate the cell viability of the developed coating solution. The proliferation of HT-29 and L929 proliferation on the CBAE coating demonstrated a slightly decreasing viability trend compared to the control. However, all viability values of the developed coating solutions were more than 90%, suggesting that all the formulated coating solutions were biocompatible and thus could be used as nontoxic materials for further application in edible packaging materials. The morphology images of HT29 and L929 on the developed coating solutions are shown in Figure 10.

### 3.4. Shelf-Life of Coated- Okra (Abelmoschus esculentus)

#### 3.4.1. Weight Loss

**Figure 11:**
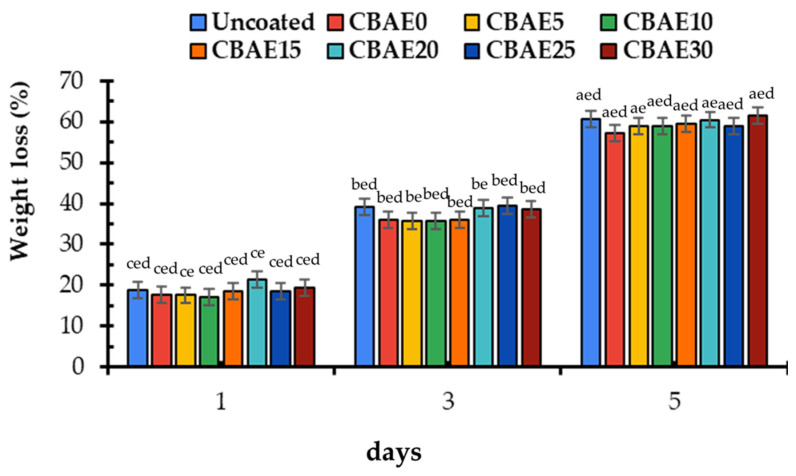
Physiological weight loss of coated and control okra during storage: CBAE0 = coated okra with 0% CBAE; CBAE5 = coated okra with 5% CBAE; CBAE10 = coated okra with 10% CBAE; CBAE15 = coated okra with 15% CBAE; CBAE20 = coated okra with 20% CBAE; CBAE25 = coated okra with 25% CBAE; and CBAE30 = coated okra with 30% CBAE, where ^a,b,c,d,e^ = different lowercase letters in same storage period indicate significant (*p* < 0.05) differences and error bars indicate ± SD.

The weight loss percentage is one of the major aspects in determining the quality and storage life of fresh products. In the current study, the weight loss percentage of the uncoated and coated okra samples significantly (*p* < 0.05) decreased after 5 days of storage, as shown in Figure 11. The uncoated samples had a slightly higher weight loss percentage than the coated okra. Considering the results, the coating treatments decreased the overall weight loss in the okra during storage in comparison to the control treatments. This might have been due to the respiration rate being retarded in the okra after the CS application in a composite solution. A similar weight loss reduction has been reported in okra coated with gum-based solutions [54].

#### 3.4.2. Firmness and Toughness Analysis

Firm texture is one of the key qualities of a fresh product. Table 2 presents the firmness values of the coated and uncoated okra samples during the investigated storage period. At the end of storage, with the uncoated okra, firmness and toughness increased by almost 100%, with the firmness and toughness of coated okra increasing by much less (16–49% and 25–90%, respectively). The firmness and toughness levels maintained with the coated okra were much better than with the uncoated sample, perhaps due to the presence of the CS. The current results are comparable with those reported by Gundewadia et al. (2018), where okra with a basil oil alginate-based coating maintained its firmness during storage at 25 °C for 8 days [55].

#### 3.4.3. Color of Coated Okra

**Figure 12:**
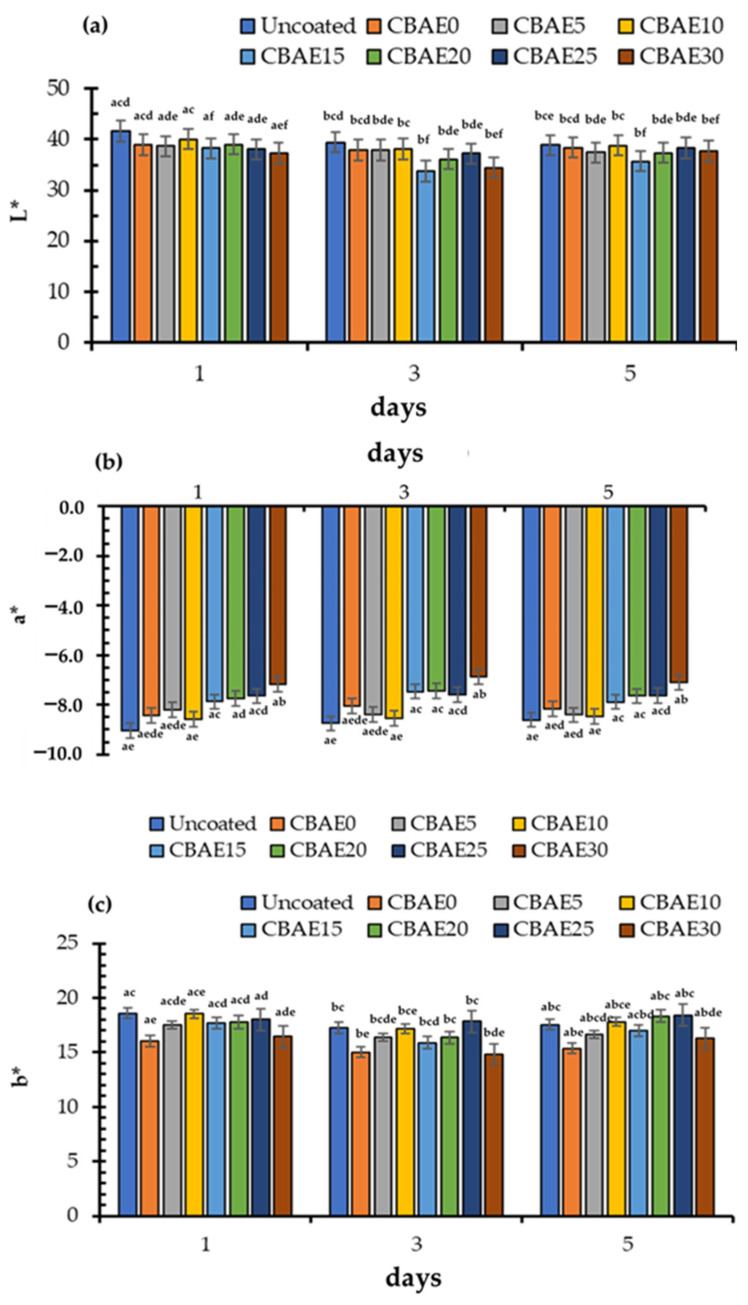
Color parameter values of coated and control okra during ambient storage; (**a**) = L*, (**b**) = a* and (**c**) = b*, where CBAE0 = coated okra with 0% CBAE; CBAE5 = coated okra with 5% CBAE; CBAE10 = coated okra with 10% CBAE; CBAE15 = coated okra with 15% CBAE; CBAE20 = coated okra with 20% CBAE; CBAE25 = coated okra with 25% CBAE; and CBAE30 = coated okra with 30% CBAE and ^a,b,c,d,e,f^ = different lowercase letters in same storage period indicate significant (*p* < 0.05) differences.

The color parameter values of the okra coated with CBAE/CS after 5 days of storage are shown in Figure 12. The L* values were in the range 35.7–41.6, showing an increasing trend over time (Figure 12a). The a* value was in the range from −6.9 to −9.0 with a slight decrease in response with an increasing CBAE concentration and storage time (Figure 12b). The b* was in the range 15–18.6 with a slight increase with an increasing CBAE concentration (Figure 12c). During storage, the coating treatment interrupted the green change with some inhibition of chlorophyll degradation, which could be explained by the lower a* values. At the end of storage, the lowest a* value was in the okra coated with 30% CBAE, followed by the 20% CBAE and the 25% CBAE coatings. However, the color change in the okra during storage also depended on other factors such as maturation, the constitution of the product, and the carotenoid content. Improvements in the color parameters in the coating formulation incorporated with the CBAE could have been due to the strong intermolecular hydrogen bonding of the hydroxyl groups between the CBAE and the CS matrix [51]. Similar results have been recorded by Shine et al. (2024), who reported that okra coated with gum could extend the product’s shelf-life [54].

## 4. Conclusions

This study successfully developed a crude bioactive ethanolic extract (CBAE) from *Spirogyra* sp. and incorporated it as a bio-filler in CS-based edible films and coatings. The CBAE, extracted via ultrasonic-assisted methods, was rich in bioactive compounds, particularly phenolics, with strong antioxidant and antibacterial properties. The formulated bio-composite films had enhanced biochemical (antioxidant activity, total phenolic content, and antimicrobial) and physical (water vapor barrier and mechanical strength) properties. Additionally, the developed coating materials were biocompatible and nontoxic, as confirmed by cell viability studies. When applied to fresh produce, the coatings effectively maintained quality attributes such as weight loss, firmness, and color retention. These findings suggest that CBAE-enhanced edible films and coatings hold potential as sustainable, active packaging materials for extending the shelf life of fresh products.

However, while the results are promising, several limitations should be noted and there are opportunities for further research. First, although the films were effective in enhancing the preservation of the quality of fresh okra, it is important to test their applicability to a broader range of fruits and vegetables, as different produce types may react differently to the films. Additionally, further exploration into the sensory qualities of the coated produce—such as taste, texture, and overall consumer acceptability—would provide a more comprehensive understanding of the potential commercial viability of these films. Furthermore, the long-term stability of the bioactive compounds within the films under various environmental conditions (such as temperature and relative humidity) has not been fully explored. In addition, studies should also aim to optimize the mechanical properties of the films to ensure their practicality for industrial-scale production. Finally, it would be useful to assess the impact of the films on food quality, particularly their sensory characteristics, to evaluate the overall acceptability and feasibility of these bioactive films in real-world applications.

## Figures and Tables

**Table 1:** Biochemical compounds in crude bioactive algal extract (CBAE).

Parameter	CBAE Result
Total phenolic content (mg GAE/g)	1.10 ± 0.31
DPPH antioxidant activity (mg GAE/g)	2.15 ± 0.09
FRAP antioxidant activity (mg GAE/g)	14.59 ± 1.61
ABTS antioxidant activity (mg GAE/g)	0.28 ± 0.07
Total Chlorophyll (mg/100 g)	61.64 ± 0.02

CBAE = crude bioactive algal extract; mg GAE = milligrams of gallic acid equivalent (GAE) per gram of sample.

**Table 2:** Effect of CBAE coatings on firmness and toughness of okra during storage.

Sample	Firmness (N)			Sample	Toughness (N.s)		
	Day1	Day3	Day5		Day1	Day3	Day5
Uncoated	5.1 ± 1.5^bAB^	6.3 ± 1.4 ^bAB^	10.5 ± 1.0 ^aAB^	Uncoated	21.6 ± 1.6 ^cA^	27.0 ± 1.9 ^bA^	46.9 ± 1.6 ^aA^
CBAE0	9.3 ± 1.9 ^bA^	7.8 ± 1.6 ^bA^	10.9 ± 1.18 ^aA^	CBAE0	28.6 ± 1.8 ^cA^	36.9 ± 2.2 ^bA^	35.7 ± 1.8 ^aA^
CBAE5	7.3 ± 0.2 ^bAB^	7.7 ± 1.3 ^bAB^	9.1 ± 0.39 ^aAB^	CBAE5	23.0 ± 0.9 ^cA^	30.8 ± 1.7 ^bA^	39.7 ± 1.8 ^aA^
CBAE10	6.7 ± 0.5 ^bAB^	9.5 ± 1.1 ^bAB^	10.0 ± 1.6 ^aAB^	CBAE10	24.0 ± 1.2 ^cA^	38.3 ± 1.5 ^bA^	35.5 ± 1.5 ^aA^
CBAE15	7.2 ± 1.1 ^bAB^	7.0 ± 0.2 ^bAB^	10.2 ± 1.9 ^aAB^	CBAE15	25.1 ± 1.4 ^cA^	33.3 ± 1.0 ^bA^	38.4 ± 1.2 ^aA^
CBAE20	7.0 ± 0.2 ^bAB^	8.7 ± 1.9 ^bAB^	9.5 ± 1.8 ^aAB^	CBAE20	22.6 ± 0.6 ^cA^	30.1 ± 1.8 ^bA^	39.5 ± 2.1 ^aA^
CBAE25	6.7 ± 0.3 ^bAB^	7.6 ± 1.8 ^bAB^	9.2 ± 0.7 ^aAB^	CBAE25	22.6 ± 2.1 ^cA^	28.6 ± 2.1 ^bA^	37.2 ± 1.0 ^aA^
CBAE30	7.4 ± 1.3 ^bB^	7.0 ± 1.8 ^bB^	8.2 ± 0.6 ^aB^	CBAE30	21.9 ± 1.5 ^cA^	32.9 ± 2.3 ^bA^	34.8 ± 1.2 ^aA^

CBAE0 = coated okra with 0% CBAE; CBAE5 = coated okra with 5% CBAE; CBAE10 = coated okra with 10% CBAE; CBAE15 = coated okra with 15% CBAE; CBAE20 = coated okra with 20% CBAE; CBAE25 = coated okra with 25% CBAE; and CBAE30 = coated okra with 30% CBAE. Data are mean ± SD values. Different lowercase letters indicate significant (*p* < 0.05) differences in storage time and different capital letters indicate significant (*p* < 0.05) differences between treatments.

## Data Availability

The original contributions presented in the study are included in the article, further inquiries can be directed to the corresponding author.
